# Arthroscopic Bankart Repair for Primary Versus Recurrent Anterior Instability in Athletes Results in Excellent Clinical Outcomes, High Rates of Return to Play, and Low Recurrence Rates

**DOI:** 10.1016/j.asmr.2021.07.011

**Published:** 2021-08-26

**Authors:** Martin S. Davey, Eoghan T. Hurley, Mohamed Gaafar, Hannan Mullett, Leo Pauzenberger

**Affiliations:** a Sports Surgery Clinic, Dublin, Ireland; b Royal College of Surgeons in Ireland, Dublin, Ireland; c National University of Ireland, Galway, Galway, Ireland

## Abstract

**Purpose:**

To compare the outcomes of athletes who have been treated for either primary or recurrent anterior shoulder instability with arthroscopic Bankart repair (ABR).

**Methods:**

A retrospective review of patients who underwent ABR for anterior shoulder instability, with a minimum of 24 months’ follow-up, was performed. Those who underwent ABR for primary instability were matched in a 1:1 ratio for age, sex, sport, and level of preoperative play to those who underwent ABR for recurrent instability. The rate, level, and timing of return to play (RTP), as well as the Shoulder Instability–Return to Sport After Injury score, were evaluated. Additionally, the recurrence rate, visual analog scale score, Subjective Shoulder Value, Rowe score, satisfaction, and whether patients would undergo the same operation again were compared.

**Results:**

After analysis of 467 patients, 100 athletes who underwent ABR for primary instability were identified and subsequently pair matched to 100 patients who underwent ABR for recurrent instability, with a mean age of 27.2 years, 87% male patients, 68% collision athletes, and a mean follow-up period of 61.9 months. There was no significant difference between the groups in the rate of RTP (80% vs 79%, *P* = .86) or RTP at the preinjury level (65% vs 65%, *P* >. 999); however, there was a significant difference in time to RTP (6.9 ± 2.9 months vs 5.9 ± 2.5 months, *P* = .02). There were no significant differences in visual analog scale score, Shoulder Instability–Return to Sport After Injury score, Subjective Shoulder Value, Rowe score, patient satisfaction, and whether patients would undergo the operation again (*P* > .05 for all). There was no difference in the rate of recurrent instability after ABR (10% vs 16%, *P* = .29).

**Conclusions:**

ABR results in excellent clinical outcomes, high rates of RTP, and low recurrence rates for both athletes with primary instability and those with recurrent instability.

**Level of Evidence:**

Level III, retrospective comparative cohort study.

Anterior shoulder instability is a common clinical issue affecting up to 2% of the general population.^1^^,^^2^ Shoulder instability occurs more commonly in the athletic population, with rates as high as 15% reported among collision athletes.^3^^,^^4^ Therefore, the treatment of anterior shoulder instability requires excellent functional outcomes because athletes often raise the primary concern of ability and timing of return to play (RTP) after injury.

Although previous literature has shown that primary instability may be managed nonoperatively, a lower rate of RTP and a 7-fold higher rate of recurrent instability limit this treatment option in athletes.^5^ Therefore, those with primary instability often elect for operative management initially over nonoperative management with the hope of a successful RTP. Arthroscopic Bankart repair (ABR) is the most commonly performed procedure for anterior shoulder instability globally, particularly in cases of soft-tissue disruption without glenoid bone loss, with excellent clinical outcomes reported after ABR.^6^ ABR has been shown to result in high rates of RTP and satisfactory functional outcomes at 10-year follow-up in both patients with primary anterior shoulder instability and those with recurrent anterior shoulder instability.^4^

The purpose of this study was to compare the outcomes of athletes who have been treated for either primary or recurrent anterior shoulder instability with ABR. Our hypothesis was that athletes undergoing ABR for primary instability would have a higher rate of RTP, better functional outcomes scores, and a lower recurrence rate when compared with those with recurrent instability.

## Methods

### Patient Selection

Having gained approval from our institutional review board, a retrospective review was carried out by 2 authors (M.S.D. and E.T.H.) to identify all patients who underwent ABR performed by a single surgeon (H.M.) between July 2012 and March 2018. The operative notes of all patients who underwent ABR for shoulder instability were analyzed with further analysis of those playing sports preoperatively. Preoperative magnetic resonance arthrography findings for each patient were analyzed to evaluate (1) the percentage of glenoid bone loss and (2) the presence or absence of off-track Hill-Sachs lesions. The inclusion criteria for this study were (1) athletes who underwent an ABR procedure and played organized sports in a league format preoperatively and (2) athletes classified as having primary or recurrent instability. The exclusion criteria for this study were (1) previous ipsilateral shoulder surgery and (2) non-athletes. Participation in collision sports was defined as participation in rugby, Gaelic Athletic Association games, hockey, or the National Football League. Subsequently, patient matching of athletes in the primary instability group (first-time dislocation) and recurrent instability group (i.e., ≥2 dislocations) based on patient demographic characteristics (age, sex, sport, level of preoperative play, and follow-up length) was performed to generate 2 comparable groups in a ratio of 1:1.

### Surgical Technique

For both procedures, all operations were performed with patients in the beach-chair position under general anesthesia. An examination under anesthesia was performed preoperatively on both shoulders to evaluate instability, range of motion, and joint laxity. Arthroscopic examination was performed through a standard posterior portal, including evaluation of the capsuloligamentous complex, and the glenoid and humerus were checked for osteochondral or osseous defects. A dynamic examination was performed to evaluate instability, laxity, and engagement of any osseous defects while moving the shoulder through its full range of motion. A probe was then used to assess the stability of the labrum and biceps anchor.

During ABR, the labrum was mobilized and the glenoid bone was freshened. The capsulolabral tissues were fixed to the glenoid rim with 2.3-mm suture anchors (Osteoraptor; Smith & Nephew, London, England) approximately from the 5-o’clock position up to the 11-o’clock position or from the 7-o’clock position up to the 1-o’clock position. All arthroscopic knots were positioned away from the joint to avoid glenohumeral irritation.

### Rehabilitation Protocol

The rehabilitation protocol was the same for all patients. Postoperatively, the shoulder was placed in a sling for 3 weeks, while non-resisted activities of daily living without excessive elevation or external rotation of the shoulder were allowed. Patients immediately began physiotherapy, which continually increased in intensity over the next 9 weeks. Return to contact in training was allowed after 12 weeks, and return to full contact and competition usually would follow within the next 3 months. In clearing an athlete to RTP, strength, range of motion, and pain were considered alongside time.

### Clinical Outcomes

Evaluation of postoperative patient-reported outcomes was carried out after a telephone survey including the rate, level, and timing of RTP, as well as the Shoulder Instability–Return to Sport After Injury (SIRSI) score.^7^ Additionally, the recurrence rate, visual analog scale (VAS) score, Subjective Shoulder Value (SSV), Rowe score, satisfaction, and whether patients would undergo the same operation again were compared.^8^^,^^9^

### Statistical Analysis

Statistical analysis was carried out using IBM SPSS Statistics for Windows software (version 22.0 [2013 release]; IBM, Armonk, NY). A power calculation was performed for the rate of recurrent instability, with an α of .05 and a power of 0.8, revealing that 200 patients were required for the study to be adequately powered. For all continuous and categorical variables, descriptive statistics were calculated. Continuous variables were reported as weighted means with estimated standard deviations, whereas categorical variables were reported as frequencies with percentages. Categorical variables were analyzed using the Fisher exact or χ^2^ test. We performed the independent or paired *t* test to compare normally distributed variables and the nonparametric Mann-Whitney *U* or Wilcoxon signed rank test to compare continuous variables. *P* < .05 was considered statistically significant.

## Results

### Patient Demographic Characteristics

Overall, 467 ABR procedures were performed by a single fellowship-trained shoulder surgeon (H.M.). After analysis, 100 athletes treated with ABR for primary instability were matched with 100 athletes treated with ABR for recurrent instability, with a mean follow-up period of 61.9 ± 20.6 months (range, 24-96 months). There were no significant differences in demographic variables between the groups. A comparison of patient demographic characteristics between the primary and recurrent instability groups is presented in Table 1.

**Table 1:** Patient Demographic Characteristics

	Primary Instability	Recurrent Instability	*P* Value
ABR, N	100	100	>.999
Age, yr	27.1 ± 7.9	27.2 ± 8.1	>.999
Follow-up, mo	61.0 ± 18.8	62.9 ± 22.4	.5166
Male sex	87 (87)	87 (87)	>.999
Collision sport	68 (68)	68 (68)	>.999
GAA	34 (34)	38 (38)	.6065
Hockey	1 (1)	2 (2)	>.999
Football	1 (1)	1 (1)	>.999
Rugby	32 (32)	27 (27)	.4890
Glenoid bone loss, %	1.7 ± 4.2	2.0 ± 4.0	.6056
Off-track Hill-Sachs lesions, %	5	10	.2828

NOTE. Data are presented as mean ± standard deviation or number (percentage).

ABR, arthroscopic Bankart repair; GAA, Gaelic Athletic Association; NFL, National Football League.

### Return to Play

Overall, there was a significant difference in the mean time to RTP between the primary and recurrent instability groups (6.9 ± 2.9 months vs 5.9 ± 2.5 months, *P* = .0207). There was no significant difference in the total rate of RTP (80% vs 79%, *P* = .8607) or the rate of return at the same level or a higher level (65% vs 65%, *P* >. 999). In patients undergoing ABR for primary instability, the reasons for not returning included shoulder injury in 11 (55%), lifestyle reasons in 6 (30%), and other injuries in 3 (15%). In those undergoing ABR for recurrent instability, the reasons for not returning included shoulder injury in 10 (47.6%), lifestyle reasons in 9 (42.9%), and other injuries in 2 (9.5%). A comparison of RTP between the primary and recurrent instability groups is presented in Table 2.

**Table 2:** Return to Play

	Primary Instability	Recurrent Instability	*P* Value
RTP	80 (80)	79 (79)	.8607
RTP at same or higher level	65 (65)	65 (65)	>.999
RTP timing, mo	6.9 ± 2.9	5.9 + 2.5	.0207
SIRSI score	64.9 ± 27.1	61.4 ± 27.2	.3631

NOTE. Data are presented as mean ± standard deviation or number (percentage).

RTP, return to play; SIRSI, Shoulder Instability–Return to Sport After Injury.

### Patient-Reported Outcomes

At final follow-up, there was no difference between patients who underwent ABR for primary instability and those who underwent ABR for recurrent instability in the reported SIRSI score (64.9 ± 27.1 vs 61.4 ± 27.2, *P* = .3631), VAS score (2.3 ± 2.3 vs 1.8 ± 1.9, *P* = .0953), SSV (84.9 ± 15.3 vs 83.6 ± 20, *P* = .6062), Rowe score (82.3 ± 19.6 vs 77.8 ± 20.5, *P* = .1142), satisfaction (86% vs 84%, *P* = .8433), or whether patients would undergo the operation again (88% vs 82%, *P* = .3222). A comparison of patient-reported outcomes between the primary and recurrent instability groups is presented in Table 3.

**Table 3:** Patient-Reported Outcomes

	Primary Instability	Recurrent Instability	*P* Value
SIRSI score	64.9 ± 27.1	61.4 ± 27.2	.3631
VAS score	2.3 ± 2.3	1.8 ± 1.9	.0953
SSV	84.9 ± 15.3	83.6 ± 20.5	.6062
Rowe score	82.3 ± 19.6	77.8 ± 13.1	.1142
Satisfied	86 (86)	84 (84)	.8433
Would undergo surgery again	88 (88)	82 (82)	.3222

NOTE. Data are presented as mean ± standard deviation or number (percentage).

SIRSI, Shoulder Instability–Return to Sport After Injury; SSV, Subjective Shoulder Value; VAS, visual analog scale.

### Recurrent Instability

Overall, 10 patients in the primary instability group and 16 patients in the recurrent instability group experienced recurrent instability after ABR (10% vs 16%, *P* = .2931), with no significant difference in rates of redislocation (6% vs 9%, *P* = .2931), subluxation (4% vs 7%, *P* = .5371), or apprehension (31% vs 34%, *P* = .7628). No other intraoperative or immediate postoperative complications occurred in our series. A comparison of recurrence between the primary and recurrent instability groups is presented in Table 4.

**Table 4:** Recurrent Instability

	Primary Instability	Recurrent Instability	*P* Value
Total recurrence	10 (10)	16 (16)	.2931
Redislocation	6 (6)	9 (9)	.4204
Subluxation	4 (4)	7 (7)	.5371
Apprehension	31 (31)	34 (34)	.7628

NOTE. Data are presented as number (percentage).

## Discussion

The most important finding of this study was that there was no difference in outcomes in athletes with either primary or recurrent anterior shoulder instability, with high rates of RTP and excellent patient-reported outcomes alongside low rates of recurrent instability. Although similar outcomes were found after ABR for both primary and recurrent instability, those athletes in the recurrent instability group managed to RTP significantly more quickly after ABR than those in the primary instability group. Thus, we can reject the hypothesis that those with recurrent instability would have inferior clinical outcomes.

The management of the athlete with primary anterior shoulder instability remains a controversial area of discussion. A wide range of recurrence rates are reported in the literature for nonoperative management of patients with primary instability, with some studies reporting rates as high as 100%.^10^, ^11^, ^12^ In a systematic review and meta-analysis, Hurley et al.^5^ reported that patients were 7-fold more likely to experience recurrent dislocations after nonoperative management when compared with ABR. Our study established that patients with failure of nonoperative treatment who undergo ABR for recurrent instability have similar clinical outcomes, as well as recurrence rates, to those treated with ABR for primary instability. However, it is worth noting that recurrent instability is not a benign event, with further bone loss and cartilage damage reported, which may warrant a more invasive procedure and yield an increased risk of long-term instability arthropathy.^13^ Thus, patients should still be counseled on their risk of recurrence before undergoing either operative or nonoperative management for primary instability.

Although patients can rely on achieving satisfactory clinical outcomes after ABR, the primary concern of athletes undergoing shoulder stabilization remains their ability to RTP acutely after treatment. Operative measures have previously been reported to show higher rates of RTP when contrasted to nonoperative management.^5^^,^^14^ Our study shows that both athletes with primary instability and those with recurrent instability reported high rates of RTP postoperatively. These findings are in keeping with findings in the previous literature; systematic reviews by Memon et al.^15^ and Ialenti et al.^16^ found that treatment of both primary and recurrent anterior shoulder instability resulted in an overall RTP rate of approximately 80%, with nearly two-thirds of patients returning at their preinjury level. However, in a study that included 271 patients at a mean of 10 years’ follow-up after ABR, Zimmermann et al.^17^ reported an RTP rate of approximately 60%, with apprehension noted in over 40% of patients. Our study showed no difference in RTP for athletes with primary instability and those with recurrent instability after ABR, despite concerns that recurrent instability may make it more difficult psychologically to RTP. The SIRSI score did not differ between the 2 groups, establishing that there was no psychological difference between the 2 cohorts.

Although many investigators have advocated nonoperative management of patients with primary instability, our study found that the use of immediate ABR in the treatment of primary instability yielded similar results when compared with patients with recurrent instability.^18^ This is of clinical interest given the finding of the aforementioned study that patients with primary instability are significantly more likely to experience recurrent dislocations after conservative management when compared with ABR.^5^

Our study found that athletes who underwent ABR for recurrent shoulder instability managed to RTP significantly more quickly than those in the primary instability group. However, in both groups, we found that athletes required approximately 6 months after ABR to RTP, regardless of their initial stabilization indication, which is slightly faster than the reported time in the literature, given that in their systematic review, Memon et al.^15^ found a mean time of approximately 9 months before RTP after ABR. Although it is still unclear why this group returned more quickly, the reason may be that the athletes had already missed a larger amount of time because of a second injury or that prior rehabilitation for their initial instability event served as a form of “pre-habilitation,” given that in our experience, we have noted that many of these athletes with prior instability continue to focus on strengthening their shoulders to prevent this second instability event.

For athletes, recurrent dislocations not only constitute lower rates of RTP and time off sport but also increase the likelihood of further soft-tissue injury, higher levels of glenoid bone loss, and further development of shoulder arthropathy.^19^, ^20^, ^21^ This holds true in particular for the young, athletic patient aged 30 years or younger with primary traumatic instability, given that a trial of nonoperative management has been reported to result in disproportionately high levels of recurrence when compared with operative management. The encouraging findings of this study strongly support the results of previous studies advising early operative treatment of primary shoulder dislocations using ABR over nonoperative management in the hope of reducing future recurrence.^5^^,^^22^

Overall, there was no significant difference in functional outcome scores between the 2 groups, with similar pain levels, SSVs, satisfaction, and willingness to undergo surgery again. Although we initially expected pain to be worse in the patients with recurrent instability owing to further damage as a result of a further instability event, this was not shown to be the case in the mid-term follow-up. However, given the high rate of instability arthropathy after ABR, this may increase with further long-term follow-up.^23^ On the basis of these findings, patients with recurrent instability can be counseled to expect similar outcomes to those with primary instability.^24^^,^^25^

### Limitations

Because the design of this study is retrospective in nature, this study has numerous limitations that are inevitable when selecting such design. This study includes 2 pair-matched groups; although matching has been performed as closely as possible, discrepancies will inherently exist. Furthermore, although all included patients were matched for age, follow-up, sex, sport, and level of sport, there were slight—albeit statistically nonsignificant—differences between the matched groups. Furthermore, this study lacks preoperative patient-reported outcome measures and laxity scores, as well as the number of preoperative dislocations and subluxations reported for each patient in the recurrent instability cohort. Finally, this study focuses on patients in a single-surgeon cohort, which—despite standardization of many factors—may limit generalizability.

## Conclusions

ABR results in excellent clinical outcomes, high rates of RTP, and low recurrence rates for both athletes with primary instability and those with recurrent instability.

## Supplementary Data

ICMJE author disclosure forms
